# Trimethoprim is associated with a greater risk of acute kidney injury and hyperkalaemia in older adults compared with other antibiotics used to treat UTIs

**DOI:** 10.1136/bmjebm-2018-110991

**Published:** 2018-06-29

**Authors:** Oghenekome Gbinigie

**Affiliations:** Nuffield Department of Primary Care Health Sciences, University of Oxford, Radcliff Primary Care Building, Woodstock Road, Oxford, England

**Commentary on**: Crellin E, Mansfield KE, Leyrat C, *et al*. Trimethoprim use for urinary tract infection and risk of adverse outcomes in older patients: cohort study. *BMJ* 2018;360:k341.

## Context

Urinary tract infection (UTI) is the most common bacterial infection in older adults.^1^ Trimethoprim is a first-line antibiotic prescribed in the UK for acuteuncomplicated UTI.^2^ Trimethoprim reduces potassium excretion in the distal nephron, which can cause elevated potassium levels.^3^ This is of particular importance in older adults, who are more likely to have comorbidities requiring prescription of additional medicines that may predispose them to hyperkalaemia, such as renin-angiotensin antagonists (RAA).

## Methods

This was a cohort study using general practice data from the Clinical Practice Research Datalink and linked Hospital Episode Statistics data in adults aged 65 and above who had received a prescription for one of five different antibiotics (trimethoprim, nitrofurantoin, cefalexin, ciprofloxacin or amoxicillin) within three days of a diagnosis of UTI in primary care. The study spanned an 18-year period from April 1997 to September 2015. The primary outcomes were acute kidney injury (AKI), hyperkalaemia and death recorded within 14 days of antibiotic initiation for UTI. The odds ratio (OR) for each outcome was calculated for each antibiotic compared with amoxicillin (chosen by the study investigators as the reference antibiotic). The authors clearly stated the questions being addressed, inclusion and exclusion criteria and methods used for statistical analysis.

## Findings

Among a cohort of 1, 191, 905 the authors identified 178, 238 individuals who received at least one antibiotic prescription for a UTI. Trimethoprim increased the odds of hyperkalaemia compared with amoxicillin (adjusted OR 2.27, 95% CI 1.49 to 3.45). Individuals prescribed trimethoprim (adjusted OR 1.72, 95% CI 1.31 to 2.24) and ciprofloxacin (adjusted OR 1.48, 95% CI 1.03 to 2.13) had increased odds of AKI compared with amoxicillin. The odds of death was not increased by prescription of any antibiotic compared with amoxicillin. There was no significant difference in the results for trimethoprim when analyses were restricted to users of RAAs.

## Commentary

This review highlights the association of prescription of trimethoprim with hyperkalaemia and AKI, but not with death, in older adults being treated for a simple UTI.

The results were adjusted for a wide range of appropriate confounders. The authors adjusted for baseline renal function, which was defined as ‘the most recent biochemical test results recorded in primary care’.^4^ Renal function declines with age.^5^ If there was a long interval between the most recent recorded renal function and renal function recorded post antibiotic prescription, it is possible that the perceived baseline renal function for some individuals may not have been a reflection of their true baseline renal function. This may have led to an overestimation of the number of cases of AKI.

Death and hyperkalaemia were determined from read codes in either primary or secondary care. However, the authors defined AKI as hospital admission with AKI. By excluding cases of AKI occurring in general practice, this may have led to an underestimation of the number of episodes of AKI.

The authors helpfully contextualise the results. While the odds of hyperkalaemia and AKI with trimethoprim prescription are increased by 127% and 72%, respectively, compared with amoxicillin prescription, the increase in the absolute risk is small. The authors state that for 1,000 UTI episodes treated with trimethoprim rather than amoxicillin, there would be one additional case of hyperkalaemia and two of AKI.^4^ These results would be very similar among those concurrently prescribed RAAs. However, among those individuals taking RAAs *and* potassium-sparing diuretics, a prescription of trimethoprim instead of amoxicillin would result in 18 additional cases of hyperkalaemia and 11 of AKI. The authors did not present ORs for the latter association in the main article.

## Implications for practice

Clinicians should be vigilant for hyperkalaemia and AKI when prescribing trimethoprim to older adults with simple UTIs. Clinicians should avoid prescribing trimethoprim to individuals taking both RAAs and potassium-sparing diuretics; if prescribed, their renal function should be monitored diligently. Trimethoprim is not associated with an increased risk of death.
